# Supplementary material for: Investigation of Thresholds for Asymmetry Indices to Represent the Visual Assessment of Single Limb Lameness by Expert Veterinarians on Horses Trotting in a Straight Line
Source: Animals (Basel). 2022 Dec 11;12(24):3498. doi: 10.3390/ani12243498 (PMC9774792; doi:10.3390/ani12243498)
Supplement: Supplementary file 1 [file animals-12-03498-s001.zip › Figure S1.pdf]

Supplementary Data 1

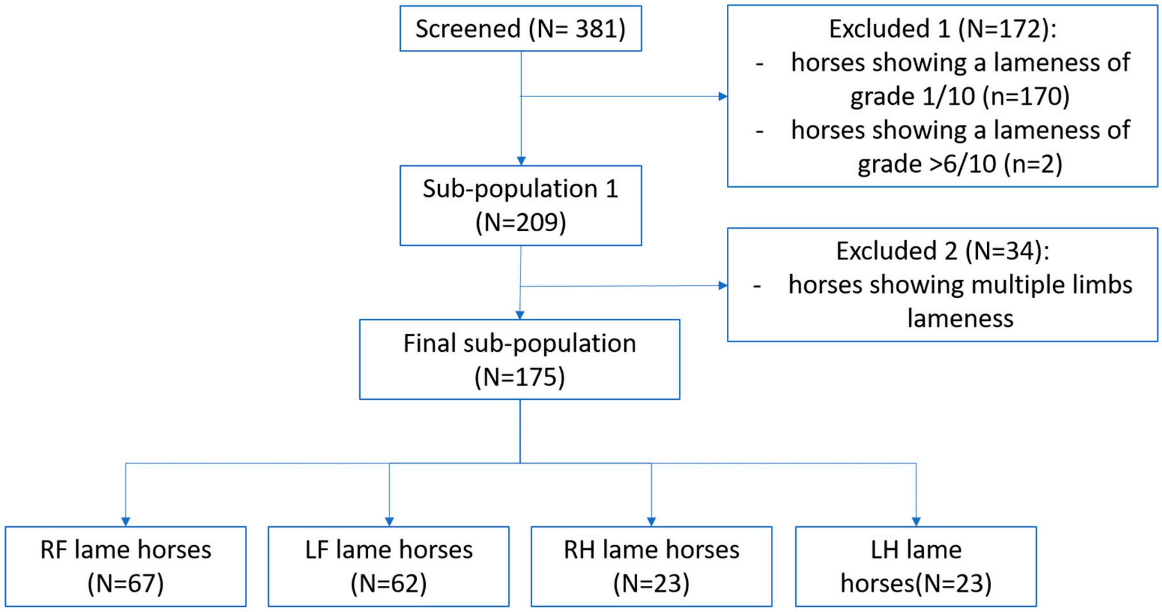

Figure S1: Flowchart of the exclusion/inclusion criteria of the screened horses, which were visually considered lame on a straight line by an expert veterinarian during his routine practice.
